# Crosstalk between Nodal/Activin and MAPK p38 Signaling Is Essential for Anterior-Posterior Axis Specification

**DOI:** 10.1016/j.cub.2011.06.048

**Published:** 2011-08-09

**Authors:** Melanie Clements, Barbara Pernaute, Francis Vella, Tristan A. Rodriguez

**Affiliations:** 1 Molecular Embryology Group, MRC Clinical Sciences Centre, Faculty of Medicine, Imperial College London, Hammersmith Hospital Campus, Du Cane Road, London W12 0NN, UK

## Abstract

Nodal/activin signaling plays a key role in anterior-posterior (A-P) axis formation by inducing the anterior visceral endoderm (AVE), the extraembryonic signaling center that initiates anterior patterning in the embryo. Here we provide direct evidence that the mitogen-activated protein kinase (MAPK) p38 regulates AVE specification through a crosstalk with the Nodal/activin signaling pathway. We show that p38 activation is directly stimulated by Nodal/activin and fails to be maintained upon inhibition of this pathway both in vivo and in vitro. In turn, p38 strengthens the Nodal signaling response by phosphorylating the Smad2 linker region and enhancing the level of Smad2 activation. Furthermore, we demonstrate that this p38 amplification loop is essential for correct specification of the AVE in two ways: first, by showing that inhibiting p38 activity in 5.5 days postcoitum embryo cultures leads to a switch from AVE to an extraembryonic visceral endoderm cell identity, and second, by demonstrating that genetically reducing p38 activity in a Nodal-sensitive background leads to a failure of AVE specification in vivo. Collectively, our results reveal a novel role for p38 in regulating the threshold of Nodal signaling and propose a new mechanism by which A-P axis development can be reinforced during early embryogenesis.

## Graphical abstract

**Graphical Abstract:**

**Figure:**
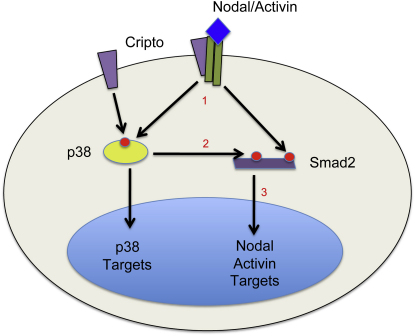

**Highlights:**

► MAPK p38 signaling is essential for specification of the A-P axis in the mouse embryo ► Activation of p38 is mediated by Nodal signaling prior to gastrulation ► Phosphorylation of the Smad2 linker region by p38 enhances Smad2 activation ► Nodal signaling requires p38 amplification to induce the anterior visceral endoderm

## Results and Discussion

### P38 Is Required for the Specification of the Anterior Visceral Endoderm

The anterior-posterior (A-P) axis of the mammalian embryo is the first of the definitive embryonic axes to be determined. The A-P axis is initiated by the induction of the anterior visceral endoderm (AVE) at the distal tip of the 5.5 days postcoitum (dpc) embryo and its migration to the prospective anterior of the embryo shortly after [1, 2]. Nodal signaling from the epiblast is thought to induce the AVE by promoting AVE-specific gene expression and by blocking inhibitory BMP signals secreted by the extraembryonic ectoderm [3–5]. It is not understood what other players are important for specification of the AVE or how the Nodal signals are interpreted within the visceral endoderm.

**Figure 1:**
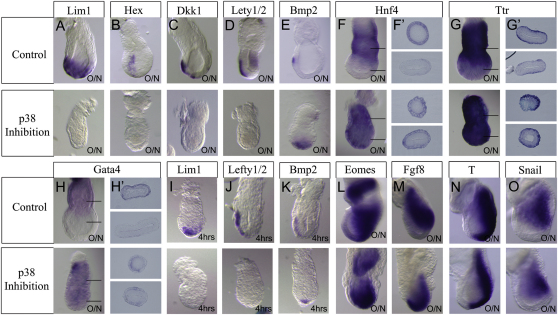
p38 Activity Is Required for AVE Induction (A–E) Expression of *Lim1*, *Hex*, *Lefty1/2*, and *Dkk1* is lost, but *Bmp2* expression is unaffected in 5.5 days postcoitum (dpc) embryos cultured overnight (O/N) in the presence of the p38 inhibitor SB203580 (n = 25, 25, 22, 25, and 32 for SB203580 treated and 19, 20, 23, 19, and 24 for controls). (F–J) The expression of the extraembryonic visceral endoderm markers *Hnf4*, *Ttr*, and *Gata4* is expanded into the embryonic visceral endoderm region after p38 inhibition in overnight cultures of 5.5 dpc embryos (n = 14, 13, and 13 for SB203580 treated and 14, 13, and 15 for controls; horizontal lines indicate the level of cross-sections shown in F′, G′, and H′). (F′–H′) Transverse sections of embryos analyzed for *Hnf4*, *Ttr*, and *Gata4* expression indicating a proximal expansion in the expression of these genes after p38 inhibition. (I–K) Inhibition for 4 hr of p38 activity in 5.5 dpc embryos abolishes *Lim1* and *Lefty1/2* expression but does not affect *Bmp2* expression (n = 8, 6, and 9 for SB203580 treated and 7, 7, and 6 for controls). (L–O) Expression of *Eomes*, *Fgf8*, *T*, and *Snail* is not decreased in overnight cultures (O/N) of 6.5 dpc embryos after p38 inhibition (n = 39, 20, 24, and 20 for SB203580 treated and 22, 18, 16, and 13 for controls).

To analyze the role of the p38 MAPK in AVE specification, we used SB203580, a specific inhibitor of the p38α and β [6], which has been used to analyze p38 function during preimplantation development [7, 8] and gastrulation [9]. When 5.5 dpc embryos were cultured overnight in the presence of SB203580, we observed that the expression of the AVE reporter *Hex*-GFP [10] was restricted to the distal tip of the embryo (see Figure S1A available online) and the expression of the AVE markers *Lim1*, *Hex*, *Dkk1*, and *Lefty1/2* was completely lost (Figures 1A–1D). In contrast, *Bmp2* expression could still be observed (Figure 1E) and the expression of the extraembryonic visceral endoderm markers *Hnf4*, *Ttr*, and *Gata4* were clearly expanded into the embryonic visceral endoderm (Figures 1F–1H′). Similar results were obtained with SB220025, a second specific inhibitor of p38α and β activity [11] (data not shown). Expression of the pluripotent epiblast marker *Oct4* and the trophoblast stem cell marker *Errβ* remained unchanged after overnight treatment of 5.5 dpc embryos with SB203580 (data not shown), and the expression of mesoderm patterning markers *Eomes*, *Fgf8*, *T*, and *Snail* was not diminished when 6.5 dpc embryos were cultured overnight in the presence of the p38 inhibitor (Figures 1L–1O). This suggests that inhibition of p38 is specifically affecting AVE specification.

To test whether p38 has a direct effect on AVE gene expression, we treated 5.5 dpc embryos with SB203580 for 4 hr. Within this time window, the expression of *Lim1* and *Lefty1/2* was lost (Figures 1I and 1J), whereas the expression of *Bmp2* could still be observed in these embryos (Figure 1K). These results suggest that p38 is directly regulating the expression of a subset of AVE genes.

### Nodal Signaling Lies Upstream of p38 Phosphorylation in the Visceral Endoderm

**Figure 2:**
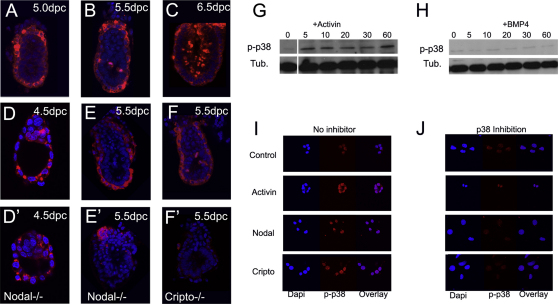
Nodal Signaling Activates p38 (A–C) Immunostaining showing that phosphorylated p38 (p-p38) is present at highest levels within the visceral endoderm between 5.0 and 6.5 dpc embryos but is also activated at high levels in mitotic cells of the epiblast from 5.5 dpc. Weak expression is observed in the epiblast at these stages. (D–F′) p-p38 expression is not affected at 4.5 dpc (D and D′) but is lost in *Nodal* (E and E′) and *Cripto* (F and F′) mutants at 5.5 dpc. (G and H) Western blot showing that activin can stimulate p38 phosphorylation within 5 min in XEN cells (G) but BMP4 cannot (H). (I) Immunofluorescence showing that activin, Nodal, and Cripto can stimulate p-p38 in XEN cells. (J) Inhibition of p38 activity by SB203580 decreases p-p38 stimulation, indicating that p38 autophosphorylation is required to enhance p38 activity.

Given the requirement for p38 activity for the correct specification of the AVE, the site of active p38 in the early embryo was investigated. At 5.0–5.5 dpc, expression of the phosphorylated (activated) form of p38 (p-p38) was highest in the cytoplasm of visceral endoderm cells, with some of these cells also showing nuclear localization. Weak expression was also observed in the cytoplasm of epiblast cells at these stages (Figures 2A and 2B). At 5.5 dpc, along with the visceral endoderm expression, mitotic cells of the epiblast were also strongly labeled by the anti-p-p38 antibody. At 6.5 dpc, this pattern was maintained, although a downregulation in the levels of p-p38 was observed within the cells of the visceral endoderm (Figure 2C). This data is consistent with a direct role for p38 in regulating AVE gene expression.

The main signaling pathway that has been shown to be responsible for AVE specification is the Nodal pathway [3]. We analyzed the expression of p-p38 in mutants for Nodal [12] and for the Nodal coreceptor Cripto [13] and found that although at 4.5 dpc the distribution of p-p38 was not significantly different between control and *Nodal* mutant embryos, p38 phosphorylation was severely downregulated or lost in both *Nodal* and *Cripto* mutants at 5.5 dpc (Figures 2D–2F′), indicating that Nodal signaling is required for p38 activation at this stage.

To further examine the requirement for Nodal activation of p38 phosphorylation, we turned to extraembryonic endoderm (XEN) cells, which are primitive endoderm stem cells and provide an in vitro model for extraembryonic endoderm development [14]. XEN cells were treated for up to 1 hr with either the TGF-β factor activin, which is also required for AVE specification [4], or with BMP4. Analysis by western blot clearly indicated that activin was capable of stimulating p38 phosphorylation within 5 min of treatment (Figure 2G). In contrast, BMP4 did not produce any increase in the levels of p38 phosphorylation (Figure 2H). Immunofluorescence also indicated that XEN cells responded to a 30 min treatment with activin, Nodal, or the Nodal coreceptor Cripto by stimulation of p38 phosphorylation (Figure 2I). This activation of p38 was dependent on its autocatalytic activity because it could be blocked using SB203580 (Figure 2J). The stimulation of p38 phosphorylation by Cripto is likely to be independent of Nodal, because this factor is not expressed in XEN cells (Figure S1G). We also observed that the activation of p38 by activin is not dependent on Alk4, 5, or 7, the type I receptors for Nodal/activin signaling, because inhibition of these receptors with SB431542 did not block p38 phosphorylation in XEN cells (Figure S1E). The findings that p38 phosphorylation can be triggered by Cripto in XEN cells and require Cripto in vivo but are not dependent on the type I TGF-β receptors indicate that Cripto plays a crucial role in p38 activation. Together, these results suggest that in the visceral endoderm, Nodal and activin are ligands that stimulate p38 activation during AVE specification and that this activation is amplified by autophosphorylation of p38.

### p38 Is Required for Maximal Nodal Signaling

**Figure 3:**
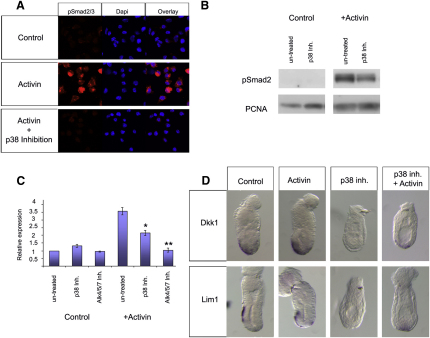
p38 Is Required for Maximal Nodal Signaling (A and B) Immunofluoresence showing that inhibition of p38 activity decreases the level of C-terminal Smad2/3 phosphorylation by activin (A) and western blot showing that inhibition of p38 activity decreases the level of C-terminal Smad2 phosphorylation by activin in XEN cells (B). (C) Luciferase assays show that inhibiting p38 activity decreases the level of stimulation by activin of the Smad2 responsive element SBE4 in XEN cells. Graph is the mean relative expression of two replicate experiments. Results are mean ± standard error of the mean of triplicate samples expressed relative to control untreated samples. ^∗^p < 0.05; ^∗∗^p < 0.001. (D) Expression of *Dkk1* and *Lim1* is partially rescued by activin in 5.5 dpc embryos cultured overnight with p38 inhibitors (n = 6 and 6 for control, 9 and 8 for activin alone, 9 and 10 for SB203580, and 10 and 10 for SB203580+activin).

We next set out to establish how p38 was affecting AVE gene expression. We asked whether p38 inhibition affected the activation of Smad2, the effector of canonical Nodal/activin signaling [15]. XEN cells were treated for 30 min with activin or Nodal in the presence of SB203580 and the levels of phosphorylated Smad2 (p-Smad2) were determined. We observed that p38 inhibition drastically reduced the overall activation by Nodal/activin of p-Smad2 and its translocation to the nucleus (Figures 3A and 3B; Figure S2F). By titrating the concentration of inhibitor used to block p38, we observed that there was a direct correlation between the levels of p-p38 and the levels of p-Smad2 (Figure S2). In contrast to this treatment, p38 inhibition did not affect the overall levels of Smad2, the levels of activation of Smad1/5/8 by BMP signaling, or the levels of activation of Erk1/2 by Fgf signaling (Figures S1B–S1D). Knocking down p38α in XEN cells using siRNA caused a 1.9-fold reduction in p38 protein and a corresponding 3.2-fold reduction in the proportion of cells showing Smad2 activation (Figure S3), confirming the specificity of the results obtained using the inhibitor. Together, these results indicate that p38 signaling is required to enhance Smad2 activation.

To analyze whether p38 inhibition also affected Smad2 target gene expression, we analyzed how p38 inhibition affected stimulation by activin of the Smad2 responsive element SBE4 [16]. This reporter contains four repeats of the Smad2 DNA binding element driving the expression of luciferase. In transient transfection assays in XEN cells, we observed that activin could efficiently stimulate expression of this reporter, whereas p38 inhibition significantly reduced this stimulation, and inhibition of the Alk4/5/7 receptors with SB43154 completely abolished it (Figure 3C). This demonstrates that p38 activity is also required for correct activation of the Nodal/activin target genes.

Next we analyzed whether p38 controls AVE specification by modulating Nodal/activin signaling. For this we cultured 5.5 dpc embryos overnight in the presence of the p38 inhibitor alone or with 100 ng/ml of activin. We observed that, as described in Figure 1, in those embryos treated with the p38 inhibitor, the expression of the AVE markers *Dkk1* and *Lim1* was completely abolished. In contrast to this, in embryos treated with the p38 inhibitor plus activin there was a partial rescue of the expression of these genes. In p38 inhibitor plus activin-treated embryos, although the expression *Dkk1* and *Lim1* was weaker than in controls and had not migrated to the prospective anterior, the expression of these genes could be clearly observed at the distal tip of the embryo (Figure 3D). This data indicates that during AVE specification p38 enhances Nodal/activin signaling but also suggests that in addition to this role, p38 is likely to have roles that are independent of Nodal/activin signaling.

The observations that p38 is required for maximal levels of Smad2 phosphorylation and for proper Nodal/activin target gene expression, together with the finding that p38 acts downstream of Nodal/activin, argue that p38 acts in a positive amplification loop for Nodal signaling.

### Smad2 Activation Is Regulated by p38

**Figure 4:**
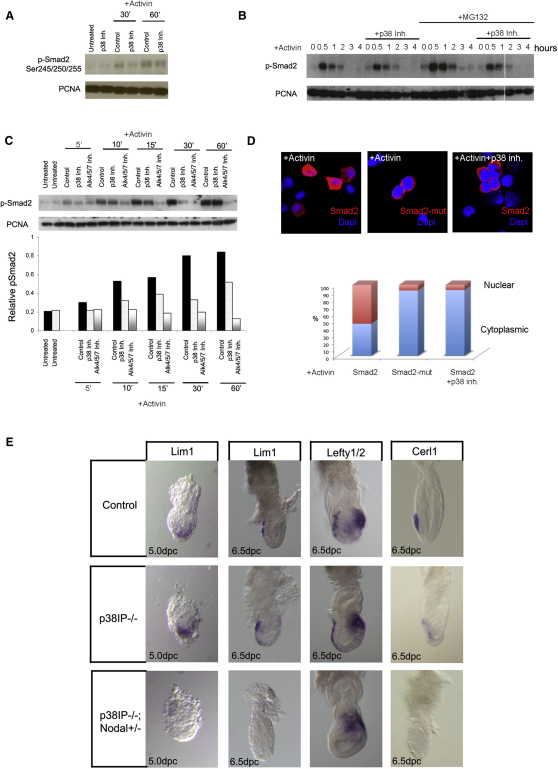
p38 Affects the Levels of Activation of Smad2 (A) Western blot showing that activin stimulates p38-dependent phosphorylation of the Smad2 linker region in XEN cells. (B) p38 inhibition can decrease C-terminal p-Smad2 phosphorylation levels even in the presence of the proteasome inhibitor MG132 and does not prolong the length of Smad2 signaling. Cells were pretreated with activin for 30 min, and then the decay of Smad2 C-terminal phosphorylation was analyzed in the presence of the type I TGF-β receptor SB431542 and in the presence or absence of the p38 inhibitor and MG132 in XEN cells. (C) Time course and quantification of activin treatment in the presence or absence of the p38 inhibitor SB203580 and of the type I TGF-β receptor inhibitor SB431542 in XEN cells show that p38 inhibition decreases activation of Smad2 C-terminal phosphorylation as early as 5 min after activin stimulation. Graph is representative of three experiments. (D) Transfection of XEN cells with Flag-Smad2 or Flag-Smad2EPSM and immunofluorescence against the Flag tag and a plot of the percentage of cells showing nuclear or cytoplasmic expression of Smad2. Mutation of the MAPK binding sites in the linker region of Smad2 inhibits nuclear accumulation after activin stimulation. (E) *Lim1* expression at 5.0 dpc and *Lim1*, *Lefty1/2*, and *Cerl1* expression at 6.5 dpc are observed in the AVE of control and p38IP mutant embryos (n = 5/5, 14/14, 11/11, and 2/2) but are reduced or lost in p38IP^−/−^; Nodal^+/−^ embryos (n = 6/7, 11/11, 2/2, and 1/1).

How is p38 enhancing Nodal signaling? The linker region of Smad2 contains four MAPK phosphorylation sites [17]; therefore, we tested whether Nodal/activin signaling led to the phosphorylation of these linker region sites. We observed that activin increased Smad2 linker region phosphorylation (Figure 4A) and that this phosphorylation was dependent on p38 activity, because inhibition by SB203580 led to a clear reduction in linker region phosphorylation (Figure 4A). This was most obvious after 30 min of activin treatment but could also be observed after 60 min of treatment. Therefore, activin via p38 caused the phosphorylation of the Smad2 linker region.

We next set out to establish at which step of the activin signaling pathway p38 is affecting Smad2 phosphorylation. Two ways in which p38 could alter Smad2 activity are by altering its rate of signaling termination or its signaling activation rate. That is, p38 could be decreasing the rate of p-Smad2 degradation or increasing the levels of p-Smad2 phosphorylation by the receptor complex. The rate of termination of Smad2 signaling can be affected in two ways, by degradation via the proteasome or by dephosphorylation by phosphatases (reviewed by [15, 18]). We analyzed whether p38 could be protecting p-Smad2 from either of these two mechanisms. XEN cells were stimulated for 30 min with activin and then signaling was blocked using the TGF-β receptor inhibitor SB431542 and the decay of C-terminal p-Smad2 was analyzed in the presence or absence of proteasome inhibitors (MG132 or lactacystin), a pan-phosphatase inhibitor or okadaic acid, an inhibitor of the PPP phosphatases that specifically act on Smad2 [19]. MG132, lactacystin, and okadaic acid caused a clear accumulation of C-terminal phosphorylated Smad2 after activin stimulation (Figure 4B; Figures S4A–S4C), indicating that degradation and dephosphorylation are regulating the overall levels of C-terminal Smad2 phosphorylation in XEN cells. We then analyzed whether p38 inhibition still caused a decrease in p-Smad2 levels in the presence of protease or phosphatase inhibition. We observed that inhibiting p38 activity in the presence of MG132, lactacystin, the pan-phosphatase inhibitor, or okadaic acid still led to a decrease in p-Smad2 phosphorylation (Figure 4B; Figures S4A–S4C), suggesting that p38 is affecting p-Smad2 levels independent of degradation via the proteasome or of phosphatase activity.

The fact that p38 is working independently of the termination of p-Smad2 signaling suggests that it must be acting upstream of these events. For this reason we analyzed whether p38 is affecting the rate of Smad2 activation by carrying out a time course of Smad2 activation kinetics in response to activin treatment and analyzing how this is affected by p38 inhibition. We observed that phosphorylation of Smad2 is stimulated as early as 5 min after activin treatment and that even at this early time point, p38 inhibition reduces the overall level of C-terminal p-Smad2 (Figure 4C). This indicates that p38 is affecting the rate of Smad2 activation and therefore it is at this step of the Nodal/activin signaling pathway that p38 is acting.

We next tested what the impact of phosphorylation of the Smad2 linker region by p38 MAPK has on the Smad2 signaling activation. For this we used a Flag-tagged Smad2 construct where the MAPK linker region phosphorylation sites have been mutated (Flag-Smad2EPSM) [20]. A critical step that occurs after Smad2 activation is its translocation to the nucleus. When we analyzed whether there was any difference in the translocation to the nucleus of Flag-Smad2EPSM with respect to Flag-Smad2, we found that although Flag-Smad2 showed efficient nuclear localization after activin stimulation (55% cells with nuclear Flag-Smad2; Figure 4D; Figure S5D), mutation of the MAPK phosphorylation sites in Smad2 nearly completely abolished this nuclear translocation (7% cells with nuclear Flag-Smad2EPSM; Figure 4D; Figure S4E). Similar results were observed when cells were transfected with the wild-type Flag-Smad2 and stimulated with activin in the presence of the p38 inhibitor (8% cells with nuclear Flag-Smad2; Figure 4D). These results demonstrate that p38 phosphorylation of the Smad2 linker region is essential for correct nuclear localization of Smad2 and to achieve high levels of Nodal/activin signaling.

### Mutation of p38IP in a Nodal-Sensitive Background Leads to a Failure to Specify the AVE

Given that we have found that p38 is critical to achieve maximal Nodal signaling, we set out to test this requirement genetically and in vivo. p38IP is a p38 interacting protein required for p38 activity and mesoderm migration during early mouse development [9]. Little is known as to how p38IP regulates p38 function and p38IP embryos correctly establish an A-P axis and specify an AVE ([9]; Figure 4). We tested whether lowering the threshold of Nodal signaling in p38IP-depleted background would affect AVE specification by generating embryos homozygous for a mutation in p38IP [9] and heterozygous for a Nodal null allele [12]. When we analyzed the expression of the AVE markers *Lim1* at 5.5 dpc and *Lim1*, *Cerl1*, and *Lefty1* at 6.5 dpc, in either control embryos or p38IP^−/−^ embryos, we observed that all three genes are appropriately expressed (Figure 4E). In contrast to this in p38IP^−/−^; Nodal^+/−^ embryos, the expression of all three AVE markers is completely lost or severely reduced in the majority of embryos analyzed (20 out of 21 embryos), showing that p38 and Nodal cooperate during AVE specification. These results indicate that p38 activity is required to enhance Nodal signaling in the embryo and therefore that the crosstalk between p38 and Nodal signaling is essential to establish the A-P axis during mouse development.

### Conclusions

Graded Nodal signaling is central to axis specification in the vertebrate embryo, and how the different thresholds of signaling are achieved determines what cell identity is specified by this pathway. At the time that the AVE is induced, high levels of expression of extracellular and intracellular inhibitors of Nodal signaling that modulate A-P axis specification have been reported [4, 21, 22]. However, no positive amplification signals that ensure that high levels of Nodal signaling are achieved in the embryo at this stage have been described. We have found that in the early embryo there is a crosstalk between p38 and the Nodal signaling pathway that enhances Nodal signaling. This crosstalk is essential for AVE induction and therefore for establishment of the A-P axis of the mouse embryo.

Our data suggest that the interaction between p38 and the Nodal/activin signaling pathways occurs at least at two levels. First, the TGF-β ligands Nodal/activin as well as the coreceptor Cripto can stimulate p38 phosphorylation and therefore activate this MAPK. Second, p38 in turn enhances Nodal/activin signaling within the visceral endoderm via phosphorylation of the Smad2 linker region to ensure maximal activation of Smad2. These results contrast with those for BMP signaling where MAPK and GSK3β phosphorylation of the Smad1 linker region decreases Smad1 stability and leads to a shorter duration of BMP signaling [17, 23]. How do we account for these differences? In the case of BMP signaling, the Smad1 linker region is phosphorylated by MAPK and GSK3β. In this case, the MAPK phosphorylation event serves as a primer for phosphorylation of the GSK3β sites [17, 23]. However, in the case of Smad2, no consensus phosphorylation sites for GSK3β exist within its linker region, and only MAPK sites can be found [15, 17]. This suggests that for Smad2, the phosphorylated MAPK sites present within its linker region do not act as primers for GSK3β phosphorylation but have a different role. We propose that by phosphorylating the Smad2 linker region, p38 increases Smad2 activation by altering its affinity either to the TGF-β receptors or to negative regulators of this pathway, such as Smad7, resulting in higher levels of p-Smad2 and therefore higher levels of Nodal/activin signaling.

Could this interaction have any relevance to other contexts where Nodal signaling is required? During gastrulation, the posterior epiblast cells undergo an epithelial to mesenchymal transition (EMT) that is essential for proper mesoderm migration from the primitive streak. Both p38IP and p38 have been shown to be required to downregulate e-cadherin in the primitive streak and therefore are essential for proper mesoderm migration to occur [9]. Nodal signaling has also been proposed to regulate EMT and control the downregulation of e-cadherin during mesoderm migration [2, 24], and we find that p38IP and Nodal genetically interact. It is therefore possible that the Nodal/p38 amplification loop may also be acting during gastrulation in the mouse embryo. In the sea urchin embryo, p38 has been shown to control Nodal expression and in this way regulate the establishment of the dorsoventral axis [25]. This suggests that the involvement of p38 in amplifying Nodal signaling may be evolutionarily conserved.

In conclusion, our work sheds light on how different thresholds of Nodal signaling are achieved within the embryo and on the consequences that these different thresholds have on cell fate decisions during early embryonic development. From a broader perspective, this work highlights a novel mechanism that enhances TGF-β signaling and therefore has important implications for the regulation of this pathway in development and disease.

## Supplemental Information

Document S1. Four Figures and Supplemental Experimental Procedures

## References

[1] Srinivas S. (2006). The anterior visceral endoderm-turning heads. Genesis.

[2] Arnold S.J., Robertson E.J. (2009). Making a commitment: Cell lineage allocation and axis patterning in the early mouse embryo. Nat. Rev. Mol. Cell Biol..

[3] Brennan J., Lu C.C., Norris D.P., Rodriguez T.A., Beddington R.S., Robertson E.J. (2001). Nodal signalling in the epiblast patterns the early mouse embryo. Nature.

[4] Yamamoto M., Beppu H., Takaoka K., Meno C., Li E., Miyazono K., Hamada H. (2009). Antagonism between Smad1 and Smad2 signaling determines the site of distal visceral endoderm formation in the mouse embryo. J. Cell Biol..

[5] Rodriguez T.A., Srinivas S., Clements M.P., Smith J.C., Beddington R.S. (2005). Induction and migration of the anterior visceral endoderm is regulated by the extra-embryonic ectoderm. Development.

[6] Cuenda A., Rouse J., Doza Y.N., Meier R., Cohen P., Gallagher T.F., Young P.R., Lee J.C. (1995). SB 203580 is a specific inhibitor of a MAP kinase homologue which is stimulated by cellular stresses and interleukin-1. FEBS Lett..

[7] Natale D.R., Paliga A.J., Beier F., D'Souza S.J., Watson A.J. (2004). p38 MAPK signaling during murine preimplantation development. Dev. Biol..

[8] Maekawa M., Yamamoto T., Tanoue T., Yuasa Y., Chisaka O., Nishida E. (2005). Requirement of the MAP kinase signaling pathways for mouse preimplantation development. Development.

[9] Zohn I.E., Li Y., Skolnik E.Y., Anderson K.V., Han J., Niswander L. (2006). p38 and a p38-interacting protein are critical for downregulation of E-cadherin during mouse gastrulation. Cell.

[10] Rodriguez T.A., Casey E.S., Harland R.M., Smith J.C., Beddington R.S. (2001). Distinct enhancer elements control Hex expression during gastrulation and early organogenesis. Dev. Biol..

[11] Kumar S., McDonnell P.C., Gum R.J., Hand A.T., Lee J.C., Young P.R. (1997). Novel homologues of CSBP/p38 MAP kinase: Activation, substrate specificity and sensitivity to inhibition by pyridinyl imidazoles. Biochem. Biophys. Res. Commun..

[12] Collignon J., Varlet I., Robertson E.J. (1996). Relationship between asymmetric nodal expression and the direction of embryonic turning. Nature.

[13] Ding J., Yang L., Yan Y.T., Chen A., Desai N., Wynshaw-Boris A., Shen M.M. (1998). Cripto is required for correct orientation of the anterior-posterior axis in the mouse embryo. Nature.

[14] Kunath T., Arnaud D., Uy G.D., Okamoto I., Chureau C., Yamanaka Y., Heard E., Gardner R.L., Avner P., Rossant J. (2005). Imprinted X-inactivation in extra-embryonic endoderm cell lines from mouse blastocysts. Development.

[15] Moustakas A., Heldin C.H. (2009). The regulation of TGFbeta signal transduction. Development.

[16] Zawel L., Dai J.L., Buckhaults P., Zhou S., Kinzler K.W., Vogelstein B., Kern S.E. (1998). Human Smad3 and Smad4 are sequence-specific transcription activators. Mol. Cell.

[17] Sapkota G., Alarcón C., Spagnoli F.M., Brivanlou A.H., Massagué J. (2007). Balancing BMP signaling through integrated inputs into the Smad1 linker. Mol. Cell.

[18] Schmierer B., Hill C.S. (2007). TGFbeta-SMAD signal transduction: Molecular specificity and functional flexibility. Nat. Rev. Mol. Cell Biol..

[19] Lin X., Duan X., Liang Y.Y., Su Y., Wrighton K.H., Long J., Hu M., Davis C.M., Wang J., Brunicardi F.C. (2006). PPM1A functions as a Smad phosphatase to terminate TGFbeta signaling. Cell.

[20] Kretzschmar M., Doody J., Timokhina I., Massagué J. (1999). A mechanism of repression of TGFbeta/ Smad signaling by oncogenic Ras. Genes Dev..

[21] Morsut, L., Yan, K.P., Enzo, E., Aragona, M., Soligo, S.M., Wendling, O., Mark, M., Khetchoumian, K., Bressan, G., Chambon, P., et al. Negative control of Smad activity by ectodermin/Tif1γ patterns the mammalian embryo. Development *137*, 2571–2578. 10.1242/dev.053801 20573697

[22] Yamamoto M., Saijoh Y., Perea-Gomez A., Shawlot W., Behringer R.R., Ang S.L., Hamada H., Meno C. (2004). Nodal antagonists regulate formation of the anteroposterior axis of the mouse embryo. Nature.

[23] Fuentealba L.C., Eivers E., Ikeda A., Hurtado C., Kuroda H., Pera E.M., De Robertis E.M. (2007). Integrating patterning signals: Wnt/GSK3 regulates the duration of the BMP/Smad1 signal. Cell.

[24] Arnold S.J., Hofmann U.K., Bikoff E.K., Robertson E.J. (2008). Pivotal roles for eomesodermin during axis formation, epithelium-to-mesenchyme transition and endoderm specification in the mouse. Development.

[25] Bradham C.A., McClay D.R. (2006). p38 MAPK is essential for secondary axis specification and patterning in sea urchin embryos. Development.

