# Supplementary material for: Crosstalk between Nodal/Activin and MAPK p38 Signaling Is Essential for Anterior-Posterior Axis Specification
Source: Curr Biol. 2011 Aug 9;21(15):1289–95. doi: 10.1016/j.cub.2011.06.048 (PMC3209556; doi:10.1016/j.cub.2011.06.048)
Supplement: Document S1. Four Figures and Supplemental Experimental Procedures [file mmc1.pdf]

Current Biology, Volume *21*

Supplemental Information

Crosstalk between Nodal/Activin and MAPK p38 Signaling Is Essential for Anterior-Posterior Axis Specification

Melanie Clements, Barbara Pernaute, Francis Vella, and Tristan A. Rodriguez

Supplemental Inventory

1. Supplemental Figures

Figure S1, related to Figure 2

Figure S2, related to Figure 3

Figure S3, related to Figure 4

Figure S4, related to Figure 4

2. Supplemental Experimental Procedures

3. Supplemental References

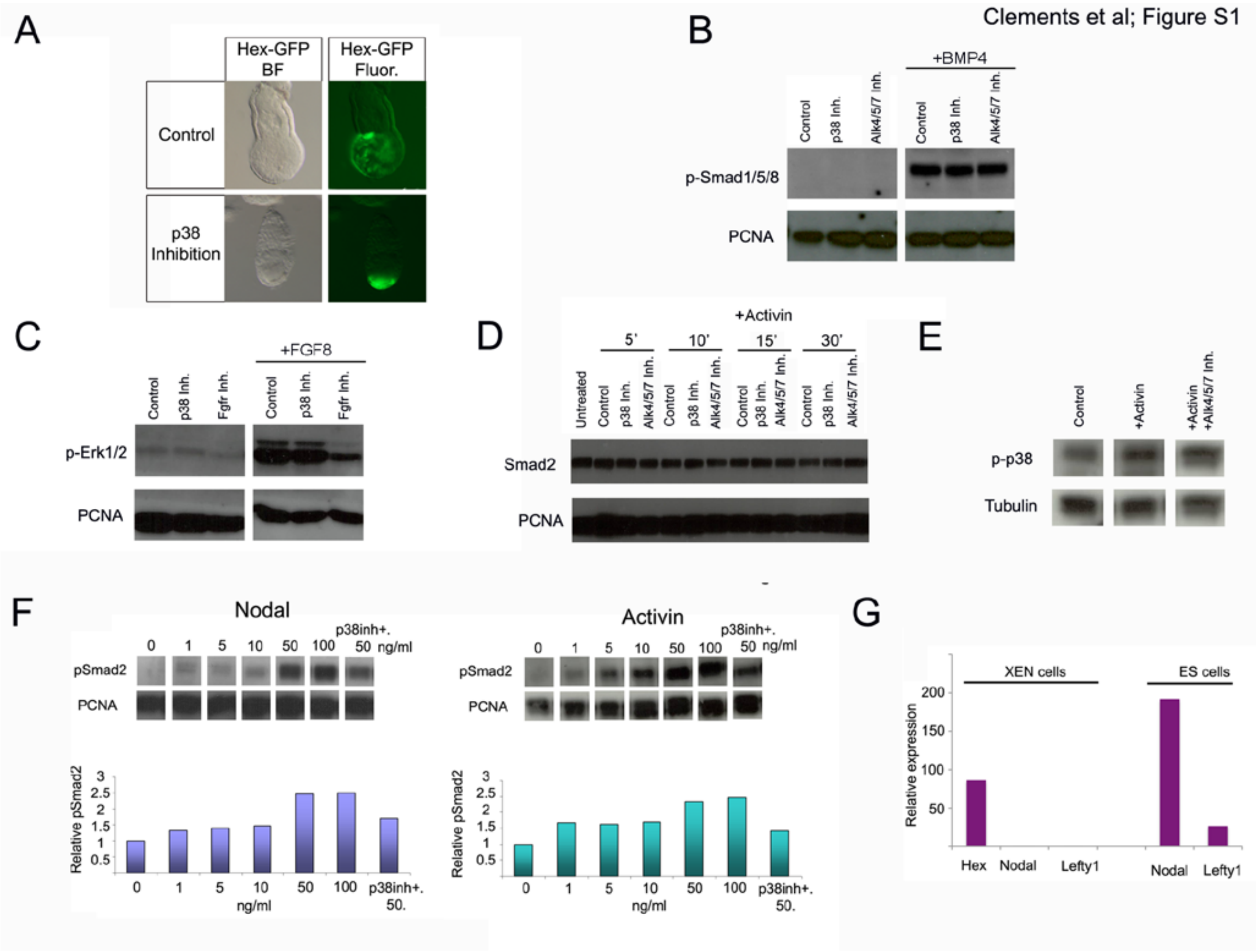

Figure S1. Nodal and Activin Specifically Trigger Smad2 Phosphorylation in XEN Cells, Related to Figure 2

(A) *Hex*-GFP expression in 5.5dpc embryos cultured overnight with SB203580 indicates that the AVE is not properly specified after inhibition of p38 activity.

(B) Western blots showing that inhibition of p38 does not affect Smad1/5/8 stimulation by BMP4 or

(C) Erk1/2 phosphorylation in response to Fgf8 in XEN cells.

(D) Time-course of Activin treatment in the presence or absence of the p38 inhibitor SB203580 and of the type I TGFβ receptor inhibitor SB431542 in XEN cells shows that neither of these treatments affects the total levels of Smad2.

(E) The type I TGFβ receptor inhibitor SB431542 does not block the increase in p38 phosphorylation triggered by Activin.

(F) Western blot and quantification of how Nodal and Activin stimulate Smad2 phosphorylation in XEN cells.

(G) Nodal and its target Lefty1 are not expressed in XEN cells but are in ESCs.

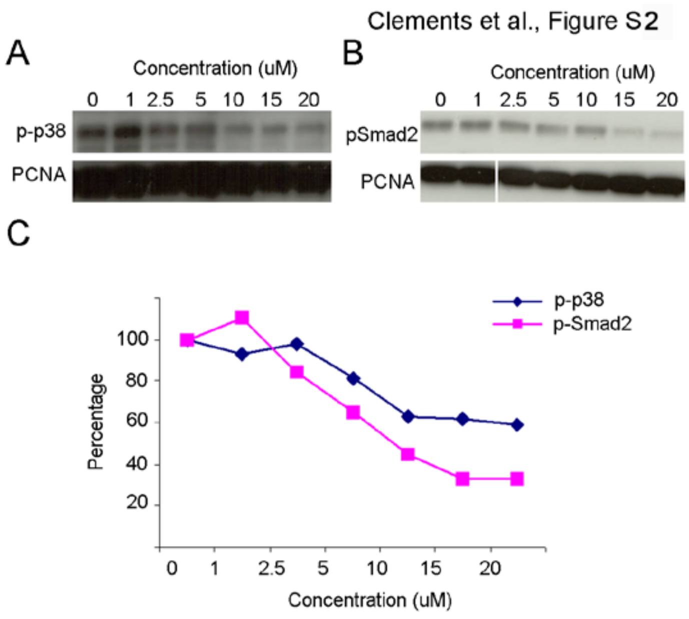

Figure S2. Inhibition of p38 Decreases Both p38 and Smad2 Phosphorylation, Related to Figure 3
Western blots and quantification of how different concentrations of the p38 inhibitor decreases p38 and Smad2 phosphorylation levels.

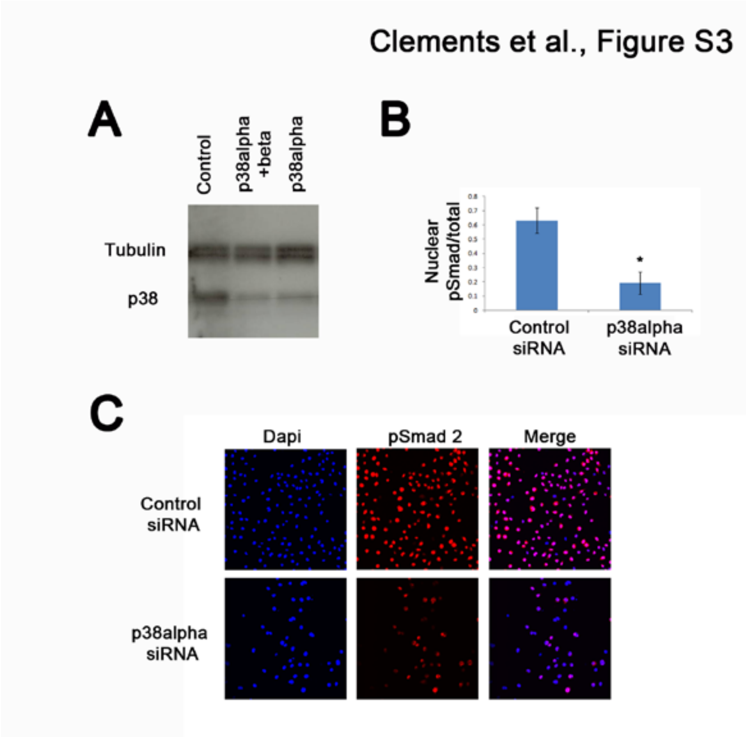

Figure S3. p38 Inhibition Decreases the Levels of Smad2 Activation, Related to Figure 4
Western blot analysis showing that siRNA against p38 leads to a decrease in p38 protein levels (A), and to decreased number of cells with phosphorylated Smad2 (B and C).

Clements et al; Figure S4

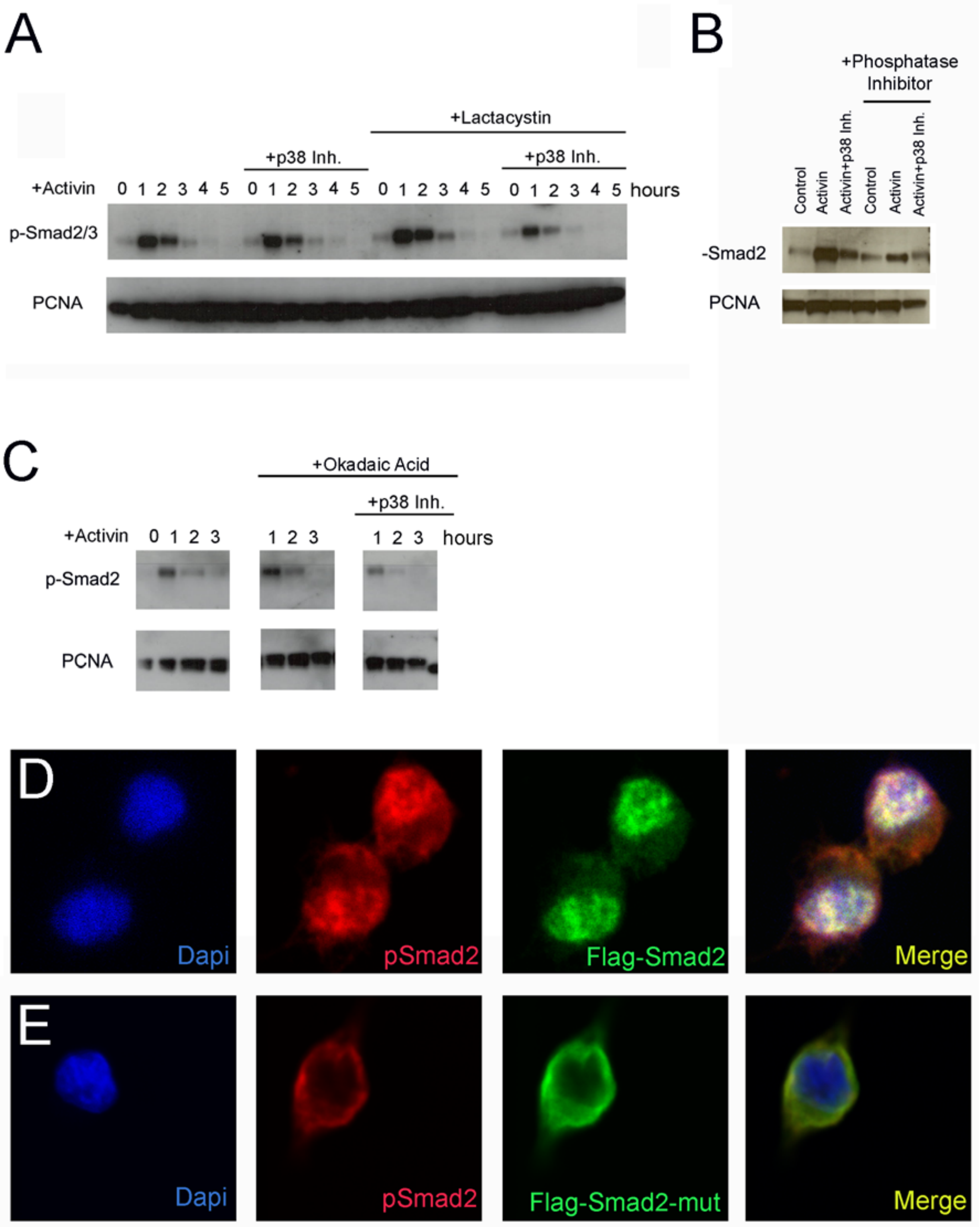

Figure S4. p38 Acts Upstream of p-Smad2 Degradation by the Proteosome or Dephosphorylation by a Phosphatase, Related to Figure 4

(A) p38 inhibition can decrease C-terminal pSmad2 phosphorylation levels even in the presence of the proteosome inhibitor lactacystin and does not prolong the length of Smad2 signalling. Cells were pre-treated with Activin for 30 minutes and then the decay of Smad2 C-terminal phosphorylation was analysed in the presence of the type I TGFβ receptor SB431542 and in the presence or absence of the p38 inhibitor and lactacystin.

(B and C) p38 inhibition can also decrease C-terminal pSmad2 phosphorylation levels in the presence of (B) a pan-phosphatase inhibitor or (C) in the presence of Okadaic acid. Cells were pre-treated with Activin for 30 minutes and then the decay of Smad2 C-terminal phosphorylation was analysed in the presence of the type I TGFβ receptor SB431542 and in the presence or absence of the p38 inhibitor and the pan-phosphatase inhibitor or okadaic acid.

(D and E) Co-localization in Activin treated XEN cells of endogenous pSmad2 and flag-Smad2 in the nucleus and of pSmad2 and flag-Smad2-EPSM (with the mutated MAPK sites) in the cytoplasm.

Supplemental Experimental Procedures

Mice and Embryos

Embryos carrying the *Hex*-GFP transgene were obtained from heterozygous *Hex*-GFP males, maintained on a mixed 129svcc, C57Bl10 and CBA background [1], crossed to CD-1 females. p38IP mice were generated from the RRK304 genetrap cell line (Sanger Centre) as previously described [2]. Genetic interaction experiments were performed using Nodal-LacZ mice [3]. All mice were maintained and treated in accordance with the Home Office's animals (Scientific Procedures) Act 1986.

Inhibitor Cultures

Embryos were cultured in 80 μl drops of DMEM:rat serum (1:1) containing either vehicle (DMSO), SB203580 (Merck, at a final concentration of 20μM) or SB220025 (Sigma, 20μM), prepared in DMSO. Drops were covered with mineral oil and embryos were incubated at 37°C in 95 % O_2 /5% CO_2 overnight (typically 16 hours) or for 4 hours. Following incubation embryos were washed three times in PBS for 5 minutes and fixed in 4 % paraformaldehyde overnight at 4 °C.

Immunohistochemistry and Whole Mount In Situ Hybridization (WMISH)

Embryos were fixed in 4% paraformaldehyde overnight at 4°C. Standard protocols for immunohistochemistry [4] and WISH [5] were used with minor modifications. For phospho-p38 a tyramide signal amplification (TSA) step was included according to manufacturers' instructions (Perkin Elmer TSA cyanine 3 kit). Antibody to phosho-p38 was used at 1:100 dilution (Cell Signalling 4631). Stained embryos were placed in glass bottom microwell dishes (MatTek Corp., USA) in a drop of 1:1 glycerol:PBS. Confocal images of the embryo were captured on a Leica DM IRB inverted confocal microscope through either a 10×/0.30 PH1 air objective or 40×/1.00 PH3 oil immersion objective.

Phospho-p38 and phosho-smad2 were visualised in XEN cells following fixation in 4% paraformaldehyde for 7 minutes and lysis in Triton-x-100 (0.1%) in PBS. A TSA step was included and cells were counterstained with mounting medium containing DAPI (Vector laboratories) to visualise nuclei. Antibodies were used at 1:100 dilution (anti phospho-p38, Cell signalling 4631; anti-phospho-Smad2/Smad3, Cell signalling 9510).

Cell Culture and Transfections

Extra-embryonic endoderm (XEN) cells were cultured in RPMI containing 15% FBS (Invitrogen 10270), β-mercaptoethanol (50μM) and sodium pyruvate (1mM) on gelatinised plates according to the methods of Kunath et al (2005). For luciferase assays, cells were seeded at a density of 1 x 10^5 cells/ml in 24-well plates and transfected with a total of 0.5μg DNA using the JetPRIME transfection reagent system according to the manufacturer's instructions (Polyplus). Cells were incubated with transfection reagent for 4 hours, fed with fresh medium and after 24 hours were washed in PBS and stimulated with Activin for 30 minutes (R&D Systems, 100ng/ml) in the presence or absence of SB203580 (20μM final concentration) or SB432125 (10μM final concentration) prepared in serum free N2B27 medium (Stem Cell Sciences). The inhibitors were maintained in the cultures until the time of luciferase expression analysis. Cell lysates were collected after a further 24 hours and luciferase activity determined (dual-luciferase reporter assay system; Promega). Plasmids used were as follows: SBE4-luc (Addgene 16495, Zawal et al, 1998); FAST-1 (Addgene 16521, Zhou et al, 1998) and the *Renilla* luciferase transfection control *pRL-SV40* (Promega).

For protein analysis XEN cells were plated on gelatinised 6-well plates in XEN cell medium at a density of 2 x 10^5 cells/ml allowed to grow overnight and washed 3 times with PBS before experimental treatments were carried out in N2B27 serum free medium.

For activation experiments cells were treated for the specified time with Activin (100ng/ml), Nodal (R&D Systems, 50ng/ml), BMP4 (R&D Systems, 50ng/ml), FGF8 (R&D Systems, 10ng/ml) or

Cripto (R&D Systems, 50ng/ml) in the presence or absence of p38 inhibitor SB203580 (20μM) or the Alk4/5/7 inhibitor SB432125 (10μM).

For degradation experiments cells were pre-treated with MG132 (50mM final concentration) or lactacystin (50mM final concentration) in the presence or absence of SB203580 (20μM) for 2 hours. Cells were stimulated with Activin (100ng/ml) for 30 minutes in the presence or absence of the proteosome inhibitor and in the presence or absence of SB203580. Activin was washed off and cells were incubated in Alk4/5/7 inhibitor SB432125 (10μM) in the presence or absence of proteosome inhibitor and in the presence or absence of SB203580. Cells lysates were collected at hourly intervals.

For phosphatase inhibition, okadaic acid (5nM final concentration) was used and cells were treated as above for the proteosome inhibition. In a second experiment Activin stimulation for 30 minutes was carried out in the presence or absence of a pan-phosphatase inhibitor (Merck 1:100 dilution) with or without p38 and Alk4/5/7 inhibition.

Indirect Immunofluorescence

XEN cells grown on gelatinised, compartmentalized glass slides in XEN cell medium were transfected as described above with CS2 Flag-Smad2 (Addgene 14042, Hata et al, 1997) or pCMV5 Flag Smad2 EPSM (Addgene 14933, Kretzschmar et al, 1999). Transfected cells were treated for 30 minutes with Activin (100ng/ml) in the presence or absence of SB203580 (20μM final concentration) or SB432125 (10μM final concentration) in N2B27 medium and the distribution of Flag protein was detected by immunofluorescence. Cells were fixed in ice-cold methanol at -20°C for 10 minutes followed by ice-cold acetone at -20°C for 1 minute and Flag protein was detected by anti-Flag antibody (Sigma 1:200 dilution) according to the manufacturer's instructions using AlexaFluor 568 goat anti rabbit antibody (Molecular probes Invitrogen 1:200) as secondary. Double immunofluorescence with anti-pSmad2 and anti-Flag antibodies was carried out to confirm co-expression of Flag and endogenous pSmad (Supplementary Fig.S4).

siRNA Knockdown

For siRNA knockdown of p38, XEN cells were transfected in N2B27 media using HiPerFect Transfection Reagent (Qiagen) according to manufacturer's instructions with siRNAs for p38 subunits. For inhibition of p38 alpha subunit a mix of 4 siRNAs against MAPK14 (Mm_Mapk14_2 FlexiTube siRNA, Mm_Mapk14_3 FlexiTube siRNA, Mm_Mapk14_4 FlexiTube siRNA, Mm_Mapk14_5 FlexiTube siRNA; Qiagen) was transfected at a final concentration of 50nM. For inhibition of p38 beta subunit a mix of 4 siRNA against MAPK11 (Mm_Mapk11_1 FlexiTube siRNA, Mm_Mapk11_2 FlexiTube siRNA, Mm_Mapk11_4 FlexiTube siRNA, Mm_Mapk11_5 FlexiTube siRNA; Qiagen) was transfected at a concentration of 50nM. For inhibition of both alpha and beta subunits together a mix of the best siRNA for each gene (all of them were tested independently) at a concentration of 50nM were used: Mm_Mapk14_4 FlexiTube siRNA for alpha inhibition plus Mm_Mapk11_4 FlexiTube siRNA for beta inhibition. A non targeting siRNA (siGENOME Non-Targeting siRNA 2, Dharmacon) was transfected at the same concentration as a negative control. 24h after transfection cells were treated with Activin A (100ng/ml) for 30 min and fixed for inmunostaining for pSmad2 and p38 as previously described. Three independent experiments were performed and a representative experiment is shown. The graph represents the average cell count +/- SEM of 5 different fields.

Protein and RNA were extracted from XEN cells following siRNA knockdown and activin treatment as above to quantify the level of p38 knockdown by siRNA transfection.

Western Blot Analysis

Cell lysates were prepared by incubation in RIPA buffer (150mM NaCl, 50mM Tris pH 8.0, 0.5% deoxycholate, 0.1% SDS, 1% NP-40) containing complete protease inhibitors (Roche) and phosphatase inhibitors (Merck). Proteins were separated by 10% SDS-PAGE (Bis-Tris Criterion^{XT} pre-cast gels, Bio-Rad). Western blots were performed using antibodies against phospho-Smad2 (1:1000, Calbiochem

566415), phospho-p38 (1:1000, Cell signalling 9211), the linker region of phospho-Smad 2 (1:1000, Cell signalling 3104), PCNA (1:1000, AbCam ab18197), a-tubulin (1:5000, AbCam ab4074), phospho-Smad1/5/8 (1:1000, Cell Signalling 9511) and phospho-Erk1/2 (1:1000, Cell Signalling 9101) according to manufacturer's datasheets.

qPCR in XEN Cells

Levels of selected AVE genes and Nodal in XEN cells were determined by qPCR. XEN cells were cultured in normal XEN media or serum free media (N2B27). mRNA extracted and qPCR performed. qPCR values were normalized against the housekeeping gene Hmbs. Values of Relative Expression are shown in comparison with Nodal levels in E14 ESCs. qPCR primers were as follows; Hmbs forward 5'ACTGGTGGAGTCTGGAGTCTAGATGGC3' and reverse 5'GCCAGGCTGATGCCCAGGTT 3'; Hex forward 5'GTCCAACGCATCCTTTTTGT3' and reverse 5'GAGGTTCTCCAACGACCACA3'; Nodal forward 5'ATGCTCAGTGGCTTGGTCTT3' and reverse 5'TCATCCTACCAACCATGCCT3'; Lefty1 forward 5'TATGTGGCCCTGCTACAACA3' and reverse 5'GCTCCATTCCGAACACTAGC3'.

Supplemental References

1. Rodriguez TA, Casey ES, Harland RM, Smith JC, Beddington RS (2001) Distinct enhancer elements control Hex expression during gastrulation and early organogenesis. Dev Biol 234: 304-316.
2. Zohn IE, Li Y, Skolnik EY, Anderson KV, Han J, et al. (2006) p38 and a p38-interacting protein are critical for downregulation of E-cadherin during mouse gastrulation. Cell 125: 957-969.
3. Collignon J, Varlet I, Robertson EJ (1996) Relationship between asymmetric nodal expression and the direction of embryonic turning. Nature 381: 155-158.
4. Nagy A, Gertsenstein M, Vintersten K, Behringer R (2003) Manipulating the Mouse Embryo. Cold Spring Harbor, New York: Cold Spring Harbor laboratory Press. 764 p.
5. Thomas P, Beddington R (1996) Anterior primitive endoderm may be responsible for patterning the anterior neural plate in the mouse embryo. Curr Biol 6: 1487-1496.
